# Galerkin-finite difference method for fractional parabolic partial differential equations

**DOI:** 10.1016/j.mex.2024.102763

**Published:** 2024-05-27

**Authors:** Md. Shorif Hossan, Trishna Datta, Md. Shafiqul Islam

**Affiliations:** Department of Applied Mathematics, University of Dhaka, Dhaka, Bangladesh

**Keywords:** Fractional PDE, Galerkin -finite difference method, Black-scholes model, Option pricing,, Galerkin-Finite Difference Method

## Abstract

The fractional form of the classical diffusion equation embodies the super-diffusive and sub-diffusive characteristics of any flow, depending on the fractional order. This study aims to approximate the solution of parabolic partial differential equations of fractional order in time and space. For this, firstly, we briefly discussed on some existing methods to solve Partial Differential Equations (PDEs) and fractional Differential Equations (DEs), then introduce a combined technique such as the Galerkin weighted residual method for the space fractional term with modified Bernoulli polynomials as basis functions, and the finite difference approximation for the time fractional term, respectively. The mathematical formulation of the proposed method is explained elaborately. Then we describe the order of convergence for the time fractional term only, as the convergence of the Galerkin method is obvious. We impose this technique on the fractional Black-Scholes model subsequently. Finally, we experimented with our proposed technique on some numerical problems. All the results are depicted in both tabular and 3D visualizations as well. We compare our results with the available methods in the literature, and our accuracy is considerable. To summarize:

• The paper introduces an approach that integrates the Galerkin weighted residual method with modified Bernoulli polynomials to handle space fractional terms, alongside employing a finite difference approximation for time fractional terms.
• The convergence analysis is focused.
• The technique is implemented on the fractional Black-Scholes model and other numerical problems, with outcomes depicted through tables and 3D visualizations.

## Graphical abstract

**Figure:**
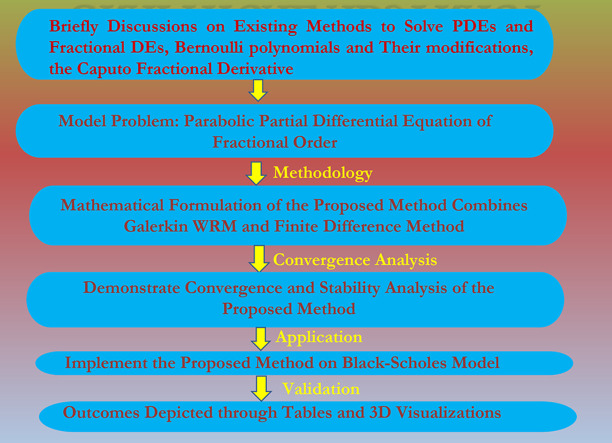

Specifications table

**Table:** 

Subject Area:	Applied Mathematics
More Specific Subject Area:	Numerical Method for Parabolic PDEs
Name of the Method:	Galerkin-Finite Difference Method
Name and Reference of Original Method:	1. F. Liu, P. Zhuang, V. Anh, I. Turner. A fractional-order implicit difference approximation for the space-time fractional diffusion equation. ANZIAM Journal, 47, C48-C68 (2005).
	2. U. Ruman, M. S. Islam. Numerical Solutions of Linear Fractional Order BVP by Galerkin Residual Method with Differentiable Polynomial. Applied and Computational Mathematics, 9(2), 20-25 (2020).
Resource Availability:	NA

## Method details

### Background

Various numerical techniques, such as finite difference method, finite element method are used in [1], [2], to solve partial differential equations (PDEs) arising in science, engineering, and other fields. Smith [3] offered detailed standard finite difference methods for various PDEs. It delves deeply into the theory behind these methods, including consistency, stability, and convergence analysis. In [4], a novel finite element approach is introduced for convection-diffusion problems, boasting second-order accuracy in time, symmetry, and unconditional stability. Douglas and Russell [5] attempted to combine finite element method or finite difference method with the method of characteristics to tackle parabolic problems.

In recent years, fractional differential equations have gained prominence across diverse fields like biology, physical science, and mechanics. Academic papers extensively explore the existence and uniqueness of solutions, examining them both analytically [6], [7] and semi-analytically [8], [9]. On the other hand, various methods such as finite difference [10], [11], finite element [12], [13], finite volume [14], spectral [15], finite difference-collocation [16], and meshless [17] techniques are employed to approximate fractional differential equations. Additionally, methods like the generalized differential transform [18], cubic B-spline wavelet collocation [19], [20], and collocation-shooting [21] are utilized for finding approximate solutions.

Complex phenomena are captured via fractional partial differential equations, like the fractional advection-dispersion equation [22]. Replacing the space derivative in the diffusion equation with a fractional order derivative leads to behaviours beyond traditional diffusion, known as sub-diffusion or super-diffusion. In [23], the collocation method utilizing traditional Legendre polynomials is implemented to achieve the numerical solution of the generalized fractional advection-diffusion equations. In [24], the authors introduced an analytical solution and presented an implicit finite difference technique capable of unconditionally stabilizing space-time fractional diffusion wave equations, even when fractional orders vary. A novel hybrid numerical technique, combining the non-standard finite difference method with Chebyshev collocation, is introduced in [25] to address fractional order diffusion equations. An analytical solution for fuzzy fractional differential equations with uncertainty has been developed in [26].

The fractional diffusion equation in space was considered by some authors in the last few decades [27], [28], [29]. However, the concern in the time-fractional diffusion equation has arisen in recent years by some researchers. The diffusion equation of fractional spatial derivative has been solved using an approximation method based on the shifted Legendre-tau concept by Abbas and Mehdi [30]. Khader presented an effective numerical approach for resolving the fractional diffusion problem [31]. It has been attempted to resolve the space fractional diffusion problem using the fourth kind of Chebyshev collocation [32]. Choi and Chung [33] have tried to approximate the solution of the diffusion equation with a nonlinear source term. They emphasized stability, existence, and convergence analysis. The fractional diffusion equation in time was considered by Liu and Huang in a half-space and whole-space in n dimension [34]. The fractional diffusion equation in both space and time derivative was discussed by Gorenflo et al. [35] and Mainardi et al. [36], and the solutions were investigated approximately. The weighted residual approach integrates diverse approximation methods for solving differential equations, evolving over fifty years. The Galerkin approach [37], [38], a prominent method within weighted residuals, is widely employed. Researchers continuously strive to discover the most effective approach that balances accuracy with computational efficiency. Recent studies have explored various techniques, including mesh-free [39] to address the fractional Black-Scholes model, Laplace homotopy perturbation [40], adaptive moving mesh [41].

From the above literature we observe that Finite difference methods, finite volume method, and some other methods are sometimes easy to implement but conditionally stable. Besides this,those methods provide the approximate values at some certain nodes (points), in which the computational costs are high and converge slowly for complex geometries. Thus, we are motivated to develop a novel technique for solving fractional parabolic partial differential equations and their practical applications.

### Method details

We provide the first Bernoulli polynomials and their modified versions to use as basis functions for the weighted residual methods throughout this paper.

#### Bernoulli Polynomials

According to [42], the Bernoulli polynomial of the nth degree can be described in terms of the range [0,1].

(1) Bn(x)=∑k=0n(nk)bkxn−k

where, $b_{k}$ are Bernoulli numbers given by,

$$b_{0}=1\;and\;b_{k}=-\int_{0}^{1}B_{k}\left(x\right)dx,\;k\geq 1$$

#### Modified Bernoulli Polynomials

Such polynomials that fulfill homogeneous boundary requirements at the end-points of the domain must be their basis function for the weighted residual technique. Usual Bernoulli polynomials (1) have failed to do so. Thus, we need to modify Bernoulli polynomials. The only way to modify Bernoulli polynomials is to subtract the Bernoulli number from the corresponding Bernoulli polynomials. Now, the Eq. (1) can be written explicitly as [43],

Bm′(x)=∑n=0m1n+1∑k=0n(−1)k(nk)(x+k)m−∑n=0m1n+1∑k=0n(−1)k(nk)km,m≥1

Since $B_{0}^{\prime }\left(x\right)$ and $B_{1}^{\prime }\left(x\right)$ do not satisfy the corresponding homogeneous boundary conditions, we redefine the basis functions over [0,1] as:

(2) $$\Phi_{m}\left(x\right)=B_{m+1}^{\prime }\left(x\right),\;\text{where}\;m\geq 1$$

Some basis functions are given below

$$\begin{array}{ccc} \Phi_{1}\left(x\right) & = & x\left(x-1\right),\;\Phi_{2}\left(x\right)=x^{3}+\frac{1}{2}\left(x-3x^{2}\right)\\ \Phi_{3}\left(x\right) & = & x^{4}-2x^{3}+x^{2},\;\Phi_{4}\left(x\right)=x^{5}+\frac{1}{6}\left(10x^{3}-15x^{4}-x\right) \end{array}$$

#### Fractional Derivatives

The idea of a fractional derivative did not emerge overnight; rather, it developed over the course of several years, beginning in 1695. Lacroix included a discussion about the derivative of non-integer order on two pages of his 700-page work from 1819. He showed that if $y=x^{a}$ then,

$$\frac{d^{\frac{1}{2}}y}{dx^{\frac{1}{2}}}=\frac{\Gamma (a+1)}{\Gamma (a+\frac{1}{2})}x^{a-\frac{1}{2}}$$

We introduce the following definition of fractional derivative that will be used throughout this paper.

##### Definition

Any function f(t) with order α>0 has the following definition for its left and right Caputo derivatives [43]:

$${}_{C}D_{a,t}^{\alpha }f\left(t\right)=D_{a,t}^{-\left(n-\alpha \right)}\left[f^{\left(n\right)}\left(t\right)\right]$$

that brings us to

$${}_{C}D_{a,t}^{\alpha }f\left(t\right)=\frac{1}{\Gamma \left(n-\alpha \right)}\int_{a}^{t}{\left(t-s\right)}^{n-\alpha -1}f^{\left(n\right)}\left(s\right)\mathrm{ds}$$

and

$${}_{C}D_{t,b}^{\alpha }f\left(t\right)=\frac{{\left(-1\right)}^{n}}{\Gamma \left(n-\alpha \right)}\int_{t}^{b}{\left(s-t\right)}^{n-\alpha -1}f^{\left(n\right)}\left(s\right)\mathrm{ds}$$

where n is an integer that is not negative and (n−1)<α<n

First, we explain the general form of model equations before discussing the Galerkin and Galerkin-Finite Difference approximations. Our model equation will often take the following form since our study takes into consideration, the space fractional diffusion equation:

(3) $$\frac{\partial u}{\partial \tau }=q\left(x\right)\frac{\partial^{\beta }u}{\partial x^{\beta }}+s\left(x,\tau \right)$$

where, q(x) represents diffusion coefficient and s(x,τ) represents source or sink term. We will solve this via the Galerkin Residual method.

In our study, we also consider the fractional diffusion equation with fractional derivative in both space and time:

(4) $$\frac{\partial^{\alpha }u}{\partial \tau^{\alpha }}=q\left(x\right)\frac{\partial^{\beta }u}{\partial x^{\beta }}+s\left(x,\tau \right)$$

To find the solutions of the Eq. (4) we introduce the Galerkin-Finite Difference approach.

#### Galerkin method for space fractional PDE

We solve the Eq. (3) with the help of the Galerkin Weighted Residual method. In this method, basis functions need to meet the homogeneous boundary requirements. And initial condition is another important step in solving any kind of partial differential equation. In this case, we select initially a trial solution. Let us consider such an approximate solution of the Eq. (3) as

(5) $$\tilde{u}\left(x,\tau \right)=\Phi_{0}\left(x,\tau \right)+\sum_{j=1}^{n}a_{j}\left(\tau \right)\Phi_{j}\left(x\right)$$

where $\Phi_{0}\left(x,\tau \right)$ satisfies the non-homogeneous part of the boundary conditions, $\Phi_{j}\left(x\right)$ denotes the basis functions which must fulfill the homogeneous boundary requirements, and the unknown parameters, $a_{j}\left(\tau \right)$ are function of time [44].

Now the residual function is

(6) $$R\left(x,\tau \right)=\frac{\partial \tilde{u}}{\partial \tau }-q\left(x\right)\frac{\partial^{\beta }\tilde{u}}{\partial x^{\beta }}-s\left(x,\tau \right)$$

Substituting the Eq. (5) into (6) we get,

(7) $$\frac{\partial \tilde{u}}{\partial \tau }=\sum_{j=1}^{n}\frac{da_{j}\left(\tau \right)}{d\tau }\Phi_{j}\left(x\right)+\frac{\partial \Phi_{0}\left(x,\tau \right)}{\partial \tau }$$

and

(8) $$\frac{\partial^{\beta }\tilde{u}}{\partial x^{\beta }}=\sum_{j=1}^{n}a_{j}\left(\tau \right)\frac{d^{\beta }\Phi_{j}\left(x\right)}{dx^{\beta }}+\frac{\partial^{\beta }\Phi_{0}\left(x,\tau \right)}{\partial x^{\beta }}$$

Then the weighted residual equation is

(9) $$\int_{0}^{1}R\left(x,\tau \right)\Phi_{i}\left(x\right)dx=0$$

where, $\Phi_{i}\left(x\right)$ are the basis functions defined in (2) for all i=1,2,...,n.

Substituting (7) and (8) into Eq. (6), then (9) leads us,

(10) $$\int_{0}^{1}\left(\sum_{j=1}^{n}\frac{da_{j}\left(\tau \right)}{d\tau }\Phi_{j}\left(x\right)+\frac{\partial \Phi_{0}\left(x,\tau \right)}{\partial \tau }-q\left(x\right)\left(\sum_{j=1}^{n}a_{j}\left(\tau \right)\frac{d^{\beta }\Phi_{j}\left(x\right)}{dx^{\beta }}+\frac{\partial^{\beta }\Phi_{0}\left(x,\tau \right)}{\partial x^{\beta }}\right)-s\left(x,\tau \right)\right)\Phi_{i}\left(x\right)dx=0$$

Now

(11) $$\begin{array}{ccc} & & \sum_{j=1}^{n}\frac{da_{j}\left(\tau \right)}{d\tau }\int_{0}^{1}\Phi_{j}\left(x\right)\Phi_{i}\left(x\right)dx+\int_{0}^{1}\frac{\partial \Phi_{0}\left(x,\tau \right)}{\partial \tau }\Phi_{i}\left(x\right)dx-\sum_{j=1}^{n}a_{j}\left(\tau \right)\\ & & \;\;\int_{0}^{1}\left(\frac{d^{\beta }\Phi_{j}\left(x\right)}{dx^{\beta }}\right)\Phi_{i}\left(x\right)q\left(x\right)dx-\int_{0}^{1}\left(\frac{\partial^{\beta }\phi_{0}\left(x,\tau \right)}{\partial x^{\beta }}\right)\Phi_{i}\left(x\right)q\left(x\right)dx-\int_{0}^{1}s\left(x,\tau \right)\Phi_{i}\left(x\right)dx=0 \end{array}$$

which can be rewritten as

(12) $$\begin{array}{ccc} & & \sum_{j=1}^{n}\frac{da_{j}\left(\tau \right)}{d\tau }\int_{0}^{1}\Phi_{j}\left(x\right)\Phi_{i}\left(x\right)dx-\sum_{j=1}^{n}a_{j}\left(\tau \right)\int_{0}^{1}\left(\frac{d^{\beta }\Phi_{j}\left(x\right)}{dx^{\beta }}\right)\Phi_{i}\left(x\right)q\left(x\right)dx\\ & & \;\;=\int_{0}^{1}\left(\frac{\partial^{\beta }\Phi_{0}\left(x,\tau \right)}{\partial x^{\beta }}\right)\Phi_{i}\left(x\right)q\left(x\right)dx-\int_{0}^{1}\frac{\partial \Phi_{0}\left(x,\tau \right)}{\partial \tau }\Phi_{i}\left(x\right)dx+\int_{0}^{1}s\left(x,\tau \right)\Phi_{i}\left(x\right)dx \end{array}$$

We can define the integrals of the above equation in terms of matrix. The dimension of the matrix depends on the number of parameters or basis functions. In matrix notation, the Eq. (12) reduces to

(13) $$\left[C\right]\;\left\{\frac{da(\tau )}{d\tau }\right\}+\left[K\right]\;\left\{a\left(\tau \right)\right\}=\left\{F\right\}$$

where,

$$\begin{array}{ccc} & & C_{ij}=\int_{0}^{1}\Phi_{j}\left(x\right)\Phi_{i}\left(x\right)dx\\ & & K_{ij}=-\int_{0}^{1}\left(\frac{\partial^{\beta }}{\partial x^{\beta }}\Phi_{j}\left(x\right)\right)\Phi_{i}\left(x\right)q\left(x\right)dx\\ & & F_{i}=\int_{0}^{1}\left(\frac{\partial^{\beta }\Phi_{0}\left(x,\tau \right)}{\partial x^{\beta }}q\left(x\right)-\frac{\partial \Phi_{0}\left(x,\tau \right)}{\partial \tau }+s\left(x,\tau \right)\right)\Phi_{i}\left(x\right)dx \end{array}$$

#### Galerkin-Finite Difference method for time-space fractional PDE

This method is a combination of discretization of time fractional terms [34], and the modified Galerkin method for space fractional terms. Now we are ready to provide the mathematical formulation for the Eq. (4) explicitly. For this, we introduce the discretized approximation of the time-fractional derivative [34] as:

$$\frac{\partial^{\alpha }u\left(x,\tau_{n+1}\right)}{\partial \tau^{\alpha }}=\frac{{\left(\delta \tau \right)}^{-\alpha }}{\Gamma (2-\alpha )}\left\{u\left(x,\tau_{n+1}\right)-u\left(x,\tau_{n}\right)+\sum_{l=1}^{n}\left(u\left(x,\tau_{n+1-l}\right)-u\left(x,\tau_{n-l}\right)\right)\left({\left(l+1\right)}^{\left(1-\alpha \right)}-l^{\left(1-\alpha \right)}\right)\right\}$$

which is equivalent to,

(14) $$\frac{\partial^{\alpha }u\left(x,\tau_{n+1}\right)}{\partial \tau^{\alpha }}=V\left[u\left(x,\tau_{n+1}\right)-u\left(x,\tau_{n}\right)+\sum_{l=1}^{n}\left(u\left(x,\tau_{n+1-l}\right)-u\left(x,\tau_{n-l}\right)\right)P_{l}\right]$$

where, $P_{l}=\left({\left(l+1\right)}^{\left(1-\alpha \right)}-l^{\left(1-\alpha \right)}\right)$, $V=\frac{{\left(\delta \tau \right)}^{-\alpha }}{\Gamma (2-\alpha )}$ and time step size as, $\delta \tau =\frac{\tau }{N}$

After applying Galerkin method, we calculate $u(x,\tau_{n})$ at different values of x, and at any specified time step, $\tau_{n}$ which is defined for n=1,2,...,N.

We choose the approximate solution as,

(15) $$\tilde{u}\left(x,\tau_{n}\right)=\Phi_{0}\left(x,\tau_{n}\right)+\sum_{i=1}^{k}a_{i}\left(\tau_{n}\right)\Phi_{i}\left(x\right)$$

where, j represents the number of parameters. Unlike as Galerkin method, $\Phi_{0}\left(x,\tau_{n}\right)$ calculates the non-homogeneous part of the boundary conditions at any specified value of time, $\tau_{n},\;a_{i}\left(\tau_{n}\right)$ are the unknown parameters whose values are needed to be determined again at the specified time, $\tau_{n}$ and $\Phi_{i}\left(x\right)$ are basis functions such as we have discussed in (2). Now we define the residual function as

$$R\left(x,\tau_{n+1}\right)=V\left[\tilde{u}\left(x,\tau_{n+1}\right)-\tilde{u}\left(x,\tau_{n}\right)+\sum_{l=1}^{n}\left(\tilde{u}\left(x,\tau_{n+1-l}\right)-\tilde{u}\left(x,\tau_{n-l}\right)\right)P_{l}\right]-q\left(x\right)\left[\frac{\partial^{\beta }\tilde{u}\left(x,\tau_{n+1}\right)}{\partial x^{\beta }}\right]-s\left(x,\tau_{n+1}\right)$$

To find a set of linear equations, we consider:

(16) $$\int_{0}^{1}R\left(x,\tau_{n+1}\right)\Phi_{j}\left(x\right)dx=0\;\;\text{where}\;j=1,2,...,k$$

or equivalently,

(17) $$\int_{0}^{1}\left(V\left[\tilde{u}\left(x,\tau_{n+1}\right)-\tilde{u}\left(x,\tau_{n}\right)+\sum_{l=1}^{n}\left(\tilde{u}\left(x,\tau_{n+1-l}\right)-\tilde{u}\left(x,\tau_{n-l}\right)\right)P_{l}\right]-q\left(x\right)\left[\frac{\partial^{\beta }\tilde{u}\left(x,\tau_{n+1}\right)}{\partial x^{\beta }}\right]-s\left(x,\tau_{n+1}\right)\right)\Phi_{j}\left(x\right)dx=0$$

After substitution of $\tilde{u}$ from Eq. (15) into Eq. (17) we get,

$$\begin{array}{ccc} & & \int_{0}^{1}(V[\left(\Phi_{0}\left(x,\tau_{n+1}\right)+\sum_{i=1}^{k}a_{i}\left(\tau_{n+1}\right)\Phi_{i}\left(x\right)\right)-\left(\Phi_{0}\left(x,\tau_{n}\right)+\sum_{i=1}^{k}a_{i}\left(\tau_{n}\right)\Phi_{i}\left(x\right)\right)\\ & & \;\;+\sum_{l=1}^{n}\left(\left(\Phi_{0}\left(x,\tau_{n+1-l}\right)+\sum_{i=1}^{k}a_{i}\left(\tau_{n+1-l}\right)\Phi_{i}\left(x\right)\right)-\left(\Phi_{0}\left(x,\tau_{n-l}\right)+\sum_{i=1}^{k}a_{i}\left(\tau_{n-l}\right)\Phi_{i}\left(x\right)\right)\right)P_{l}]\\ & & \;\;-q\left(x\right)\left[\frac{\partial^{\beta }\left(\Phi_{0}\left(x,\tau_{n+1}\right)+\sum_{i=1}^{k}a_{i}\left(\tau_{n+1}\right)\Phi_{i}\left(x\right)\right)}{\partial x^{\beta }}\right]-s\left(x,\tau_{n+1}\right))\Phi_{j}\left(x\right)dx=0 \end{array}$$

or,

$$\begin{array}{ccc} & & V[\int_{0}^{1}\left(\left(\Phi_{0}\left(x,\tau_{n+1}\right)+\sum_{i=1}^{k}a_{i}\left(\tau_{n+1}\right)\Phi_{i}\left(x\right)\right)\Phi_{j}\left(x\right)\right)dx-\int_{0}^{1}\left(\left(\Phi_{0}\left(x,\tau_{n}\right)+\sum_{i=1}^{k}a_{i}\left(\tau_{n}\right)\Phi_{i}\left(x\right)\right)\Phi_{j}\left(x\right)\right)dx\\ & & \;\;+\;\sum_{l=1}^{n}(\int_{0}^{1}\left(\left(\Phi_{0}\left(x,\tau_{n+1-l}\right)+\sum_{i=1}^{k}a_{i}\left(\tau_{n+1-l}\right)\Phi_{i}\left(x\right)\right)\Phi_{j}\left(x\right)\right)dx-\int_{0}^{1}((\Phi_{0}\left(x,\tau_{n-l}\right)\\ & & \;\;+\;\sum_{i=1}^{k}a_{i}\left(\tau_{n-l}\right)\Phi_{i}\left(x\right))\Phi_{j}\left(x\right))dx)P_{l}]-\int_{0}^{1}q\left(x\right)[\frac{\partial^{\beta }(\Phi_{0}\left(x,\tau_{n+1}\right)}{\partial x^{\beta }}+\sum_{i=1}^{k}a_{i}\left(\tau_{n+1}\right)\frac{\partial^{\beta }\left(\Phi_{i}\left(x\right)\right)}{\partial x^{\beta }}]\Phi_{j}\left(x\right)dx\\ & & \;\;-\int_{0}^{1}s\left(x,\tau_{n+1}\right)\Phi_{j}\left(x\right)dx=0 \end{array}$$

or,

(18) $$\begin{array}{cc} & V[\int_{0}^{1}(\left(\Phi_{0}\left(x,\tau_{n+1}\right)-\Phi_{0}\left(x,\tau_{n}\right)\right)\Phi_{j}\left(x\right)dx+\sum_{i=1}^{k}\left(a_{i}\left(\tau_{n+1}\right)-a_{i}\left(\tau_{n}\right)\right)\int_{0}^{1}\Phi_{i}\left(x\right)\Phi_{j}\left(x\right)dx\\ & \;+\sum_{l=1}^{n}(\int_{0}^{1}(\left(\Phi_{0}\left(x,\tau_{n+1-l}\right)-\Phi_{0}\left(x,\tau_{n-l}\right)\right)\Phi_{j}\left(x\right)dx+\sum_{i=1}^{k}\left(a_{i}\left(\tau_{n+1-l}\right)-a_{i}\left(\tau_{n-l}\right)\right)\\ & \;\times \int_{0}^{1}\Phi_{i}\left(x\right)\Phi_{j}\left(x\right)dx)P_{l}]-\int_{0}^{1}q\left(x\right)(\frac{\partial^{\beta }\left(\Phi_{0}\left(x,\tau_{n+1}\right)\right)}{\partial x^{\beta }})\Phi_{j}\left(x\right)dx-\sum_{i=1}^{k}a_{i}\left(\tau_{n+1}\right)\\ & \;\times \int_{0}^{1}q\left(x\right)\frac{\partial^{\beta }\left(\Phi_{i}\left(x\right)\right)}{\partial x^{\beta }}\Phi_{j}\left(x\right)dx-\int_{0}^{1}s\left(x,\tau_{n+1}\right)\Phi_{j}\left(x\right)dx=0 \end{array}$$

We evaluate the values of unknown parameters, $a_{i}\left(\tau_{n}\right)$ after substituting the value of n in Eq. (18). For example, if we substitute n=0, we obtain linear equations at τ=δτ. Solving those equations we find out an approximate solution at a specified time value as a function of x such as $\tilde{u}\left(x,\delta \tau \right)$. Repeating the steps for n=1,2,...,N, we will be able to calculate the values of u(x,τ) at different x for any value of time. We express the integrals in terms of matrix and in matrix notation,

(19) $$V[\left[G\right]\left\{a\left(\tau_{n+1}\right)-a\left(\tau_{n}\right)\right\}\;+\sum_{l=1}^{n}(\left\{H\right\}+\left[G\right]\left\{a\left(\tau_{n+1-l}\right)-a\left(\tau_{n-l}\right)\right\})P_{l}]=\left[K\right]\left\{a\left(\tau_{n+1}\right)\right\}+\left\{M\right\}$$

where

$$\begin{array}{cc} G_{ij} & =\int_{0}^{1}\Phi_{i}\left(x\right)\Phi_{j}\left(x\right)dx\\ H_{j} & =\int_{0}^{1}(\left(\Phi_{0}\left(x,\tau_{n+1-l}\right)-\Phi_{0}\left(x,\tau_{n-l}\right)\right)\Phi_{j}\left(x\right)dx\\ K_{ij} & =\int_{0}^{1}q\left(x\right)\left(\frac{\partial^{\beta }}{\partial x^{\beta }}\Phi_{i}\left(x\right)\right)\Phi_{j}\left(x\right)dx\\ M_{j} & =\int_{0}^{1}(q\left(x\right)\frac{\partial^{\beta }\left(\Phi_{0}\left(x,\tau_{n+1}\right)\right)}{\partial x^{\beta }}-V\left(\Phi_{0}\left(x,\tau_{n+1}\right)-\Phi_{0}\left(x,\tau_{n}\right)\right)+s\left(x,\tau_{n+1}\right))\Phi_{j}\left(x\right)dx \end{array}$$

## Method validation

To simplify the convergence analysis of the Galerkin-Finite Difference method, we consider the following fractional diffusion equation in both space and time but without a source term and a constant diffusion coefficient (K).

(20) $$\frac{\partial^{\alpha }u}{\partial \tau^{\alpha }}=K\frac{\partial^{\beta }u}{\partial x^{\beta }}$$

If we write the residual function setting to zero we get,

(21) $$V\left[\tilde{u}\left(x,\tau_{n+1}\right)-\tilde{u}\left(x,\tau_{n}\right)+\sum_{i=1}^{n}\left(\tilde{u}\left(x,\tau_{n+1-i}\right)-\tilde{u}\left(x,\tau_{n-i}\right)\right)p_{i}\right]=K\left[\frac{\partial^{\beta }\tilde{u}\left(x,\tau_{n+1}\right)}{\partial x^{\beta }}\right]$$

Particularly, for n=0, $\left(\tau_{1}=\delta t\right)$ the Eq. (21) can be written as,

(22) $$\tilde{u}\left(x,\tau_{1}\right)-\frac{K}{V}\left[\frac{\partial^{\beta }\tilde{u}\left(x,\tau_{1}\right)}{\partial x^{\beta }}\right]=p_{0}\tilde{u}\left(x,\tau_{0}\right)$$

and, in general, we get the following equation,

(23) $$\tilde{u}\left(x,\tau_{n+1}\right)-\frac{K}{V}\left[\frac{\partial^{\beta }\tilde{u}\left(x,\tau_{n+1}\right)}{\partial x^{\beta }}\right]=\left(1-p_{1}\right)\tilde{u}\left(x,\tau_{n}\right)+p_{n}\tilde{u}\left(x,\tau_{0}\right)+\sum_{i=1}^{n-1}\left(p_{i}-p_{i+1}\right)\tilde{u}\left(x,\tau_{n-i}\right)$$

Let us introduce error as, $\epsilon \left(x_{m},\tau_{n}\right)=\epsilon_{m}^{n}=u_{m}^{n}-{\tilde{u}}_{m}^{n}$ with $\epsilon^{n}={\left(\epsilon_{1}^{n},\epsilon_{2}^{n},...,\epsilon_{M-1}^{n}\right)}^{T}$ and $\epsilon^{0}=0$. In our study we introduce, $\frac{\partial^{\beta }}{\partial x^{\beta }}=D^{\beta }$. After substitution of the approximate solution into Eq. (22) and (23) we get,

$$\epsilon_{m}^{1}-\frac{K}{V}\left[D^{\beta }\left(\epsilon_{m}^{1}\right)\right]=\epsilon_{m}^{0}+R_{m}^{1}$$

$$\text{and,}\;\epsilon_{m}^{n+1}-\frac{K}{V}\left[D^{\beta }\left(\epsilon_{m}^{n+1}\right)\right]=\left(1-p_{1}\right)\epsilon_{m}^{n}+p_{n}\epsilon_{m}^{0}+\sum_{i=1}^{n-1}\left(p_{i}-p_{i+1}\right)\epsilon_{m}^{n-i}+R_{m}^{n+1}$$

In this case, the value of m remains the same but n varies from 1 to N−1. And we are going to use, $|R_{m}^{n}|\leq C\left(\delta \tau \right)\;\text{where,}\;n=1,2,...,N\;\text{and}\;m=1,2,...,M-1$

Now, through letting $∥\epsilon^{1}{∥}_{\infty }=\left|\epsilon_{j}^{1}\right|\;\text{where,}\;x_{j}=max\left(x_{m}\right)$, we can write,

$$|\epsilon_{j}^{1}\left|\leq \right|\epsilon_{j}^{1}|-\frac{K}{V}[D^{\beta }\left(\right|\epsilon_{j}^{1}\left|)\right]$$

$$\;\leq |\epsilon_{j}^{1}-\frac{K}{V}\left[D^{\beta }\left(\epsilon_{j}^{1}\right)\right]|$$

$$\;=|\epsilon_{j}^{0}+R_{j}^{1}|$$

Since, $\epsilon^{0}=0\;\;\text{and,}\;|R_{j}^{1}|\leq C\left(\delta \tau \right)\;\text{then,}\;\;∥\epsilon^{1}{∥}_{\infty }\leq C\left(\delta \tau \right)$ For the next part of the proof we consider the following:

$$∥\epsilon^{i}{∥}_{\infty }\leq Cp_{i-1}^{-1}\left(\delta \tau \right),\;p_{i}^{-1}\geq p_{n}^{-1}\;\;\text{and}\;\;∥\epsilon^{n+1}{∥}_{\infty }=\left|\epsilon_{j}^{n+1}\right|;\;\;\text{where,}\;x_{j}=max\left(x_{m}\right).$$

$$\begin{array}{cc} \text{Now,}\;|\epsilon_{j}^{n+1}| & \leq |\epsilon_{j}^{n+1}|-\frac{K}{V}[D^{\beta }\left(\right|\epsilon_{j}^{n+1}|)]\;\;\;\;\;\;\;\;\;\;\;\;\\ & \leq |\epsilon_{j}^{n+1}-\frac{K}{V}\left[D^{\beta }\left(\epsilon_{j}^{n+1}\right)\right]|\\ & =|\left(1-p_{1}\right)\epsilon_{j}^{n}+p_{n}\epsilon_{j}^{0}+\sum_{i=1}^{n-1}\left(p_{i}-p_{i+1}\right)\epsilon_{j}^{n-i}+R_{j}^{n+1}|\\ & \leq \left(1-p_{1}\right)|\epsilon_{j}^{n}|+\sum_{i=1}^{n-1}\left(p_{i}-p_{i+1}\right)|\epsilon_{j}^{n-i}\left|+\right|R_{j}^{n+1}|\\ & \leq \left\{\left(1-p_{1}\right)p_{n-1}^{-1}+\sum_{i=1}^{n-1}\left(p_{i}-p_{i+1}\right)p_{n-i-1}^{-1}\right\}C\left(\delta \tau \right)+\left|R_{j}^{n+1}\right| \end{array}$$

So, $∥\epsilon^{n+1}{∥}_{\infty }\leq p_{n}^{-1}\left\{\left(1-p_{1}\right)+\sum_{i=1}^{n-1}\left(p_{i}-p_{i+1}\right)+p_{n}\right\}C\left(\delta \tau \right)=p_{n}^{-1}C\left(\delta \tau \right)$. Therefore, the convergence order of the scheme is given by

$$|{\tilde{u}}_{m}^{n}-u\left(x_{m},\tau_{n}\right)|\leq C\left(\delta \tau \right)\;\text{where,}\;n=1,2,...,N\;\text{and}\;m=1,2,...,M-1.$$

Throughout this, we have exploited C as a positive constant with (0.5<C<2) depending on the problem. The following theorem is derived for the case, 0<α<1. Based on the above discussion and literature [[34], [45]], we can state the following similar theorem:

**Theorem:** Consider the approximate solution as $\tilde{u}\left(x_{m},\tau_{n}\right)$ of the exact solution $u(x_{m},\tau_{n})$, and for simplification, we choose, $\tilde{u}\left(x_{m},\tau_{n}\right)={\tilde{u}}_{m}^{n}$ and $u\left(x_{m},\tau_{n}\right)=u_{m}^{n}$. Then there is always a positive constant C such that,

$$|{\tilde{u}}_{m}^{n}-u_{m}^{n}|\leq C\left(\delta \tau \right)\;\text{where,}\;n=1,2,...,N\;\text{and}\;m=1,2,...,M-1.$$

For stability analysis, we insert (14) into (20) to get

(24) $$V\left[u\left(x,\tau_{n+1}\right)-u\left(x,\tau_{n}\right)+\sum_{i=1}^{n}\left(u\left(x,\tau_{n+1-i}\right)-u\left(x,\tau_{n-i}\right)\right)p_{i}\right]=K\left[\frac{\partial^{\beta }u\left(x,\tau_{n+1}\right)}{\partial x^{\beta }}\right]$$

On using (15), (21) and (24) we can write

(25) $$\begin{array}{cc} & V[u\left(x,\tau_{n+1}\right)-\tilde{u}\left(x,\tau_{n+1}\right)-\left(u\left(x,\tau_{n}\right)-\tilde{u}\left(x,\tau_{n}\right)\right)+\sum_{i=1}^{n}\left(u\left(x,\tau_{n+1-i}\right)-\tilde{u}\left(x,\tau_{n+1-i}\right)-\left(u\left(x,\tau_{n-i}\right)-\tilde{u}\left(x,\tau_{n-i}\right)\right)\right)p_{i}]\\ & =K\left[\frac{\partial^{\beta }u\left(x,\tau_{n+1}\right)}{\partial x^{\beta }}-\frac{\partial^{\beta }\tilde{u}\left(x,\tau_{n+1}\right)}{\partial x^{\beta }}\right] \end{array}$$

Let us now define the error term at n-th time step as:

$$E_{m}^{n}=u\left(x_{m},t_{n}\right)-\tilde{u}\left(x_{m},t_{n}\right)\;\text{where,}\;n=0,1,2,...,N\;\text{and}\;m=0,1,2,...,M.$$

Inserting the above error term in Eq. (25) for any m-th space step we can write,

(26) $$V\left[E_{m}^{n+1}-E_{m}^{n}+\sum_{i=1}^{n}\left(E_{m}^{n+1-i}-E_{m}^{n-i}\right)p_{i}\right]=K\frac{\partial^{\beta }}{\partial x^{\beta }}\left(E_{m}^{n+1}\right).$$

Now for n=0, the Eq. (26) becomes

(27) $$V\left[E_{m}^{1}-E_{m}^{0}\right]=K\frac{\partial^{\beta }}{\partial x^{\beta }}\left(E_{m}^{1}\right).$$

We consider $L_{\infty }$ norm of the error defined as $∥E^{1}{∥}_{\infty }=\left|E_{j}^{1}\right|,\;\text{where}\;x_{j}=max\left(x_{m}\right)$, and (27) leads us

(28) $$∥E^{1}{∥}_{\infty }=|E_{j}^{1}\left|\leq \right|E_{j}^{1}|-\frac{K}{V}(\frac{\partial^{\beta }}{\partial x^{\beta }}|E_{j}^{1}\left|)=\right|E_{j}^{0}|\leq ∥E^{0}{∥}_{\infty }.$$

For n=1, the Eq. (26) reduces to

(29) $$V\left[E_{m}^{2}-E_{m}^{1}+\left(E_{m}^{1}-E_{m}^{0}\right)p_{1}\right)]=K\frac{\partial^{\beta }}{\partial x^{\beta }}\left(E_{m}^{2}\right).$$

Using the notation $∥E^{2}{∥}_{\infty }=\left|E_{j}^{2}\right|$, we obtain.

(30) $$\begin{array}{cc} & ∥E^{2}{∥}_{\infty }=|E_{j}^{2}\left|\leq \right|E_{j}^{2}|-\frac{K}{V}(\frac{\partial^{\beta }}{\partial x^{\beta }}|E_{j}^{2}\left|)=\right|\left(1-p_{1}\right)E_{j}^{1}+p_{1}E_{j}^{0}|\\ & \leq \left(1-p1\right)|E_{j}^{0}|+p_{1}|E_{j}^{0}|\leq ∥E^{0}{∥}_{\infty } \end{array}$$

Similarly, by using method of induction we can show that for any l-th time step,

(31) $$∥E^{l+1}{∥}_{\infty }\leq {∥E^{0}∥}_{\infty }$$

Thus, we may conclude that the finite difference of the time fractional term is unconditionally stable, and details are available in [34].

Now, we consider four problems, including two applications that appear in Financial Mathematics. Here we define an absolute error, $L_{\infty }$-norm and order of convergence, respectively, as follows,

$$\text{Absolute}\text{Error}=|\tilde{u}\left(x,t\right)-u\left(x,t\right)|$$

$$L_{\infty }\text{-norm}=max\left(A_{i}\right),\;\text{where}\;A_{i}\;\text{are}\;\text{the}\;\text{absolute}\;\text{errors.}$$

$$\text{Order}\;\text{of}\;\text{Convergence,}\;C\left(\delta t\right)=\frac{e_{1}}{e_{2}}$$

where, $e_{1}$ and $e_{2}$ represents $L_{\infty }$- norm for different time step size.

**Problem-1:** We consider the problem defined in Eq. (3) and is taken from [32], on a specified domain, 0<x<1, diffusion coefficient $q\left(x\right)=\Gamma \left(1.2\right)x^{1.8}$ and the source term $s\left(x,t\right)=3x^{2}\left(2x-1\right)e^{-t}$ such that

(32) $$\frac{\partial u(x,t)}{\partial t}=q\left(x\right)\frac{\partial^{1.8}u\left(x,t\right)}{\partial x^{1.8}}+s\left(x,t\right)$$

with the boundary and the initial conditions

$$u\left(0,t\right)=0,\;u\left(1,t\right)=0\;\text{where,}\;t>0\;\text{and,}\;u\left(x,0\right)=x^{3}.$$

The exact solution is:

$$u\left(x,t\right)=x^{2}\left(1-x\right)e^{-t}$$

Using the mathematical formulation described for space fractional PDE with two parameters let us approximate the solution in the form:

(33) $$\tilde{u}\left(x,t\right)=\sum_{i=1}^{2}a_{i}\left(t\right)\Phi_{i}\left(x\right).$$

We obtain the following residual function:

(34) $$R\left(x,t\right)=\frac{\partial \tilde{u}\left(x,t\right)}{\partial t}-\left(q\left(x\right)\frac{\partial^{1.8}\tilde{u}\left(x,t\right)}{\partial x^{1.8}}+s\left(x,t\right)\right)$$

and the residual equation force us

(35) $$\int_{0}^{1}R\left(x,t\right)\Phi_{j}\left(x\right)dx=0\;\text{where,}\;j=1,2$$

$$\begin{array}{cc} \text{or,} & \;\sum_{i=1}^{2}\frac{da_{i}\left(t\right)}{dt}\int_{0}^{1}\Phi_{i}\left(x\right)\Phi_{j}\left(x\right)dx-\sum_{i=1}^{2}a_{i}\left(t\right)\int_{0}^{1}\left(\frac{d^{1.8}}{dx^{1.8}}\Phi_{i}\left(x\right)\right)\Phi_{j}\left(x\right)q\left(x\right)dx-\int_{0}^{1}s\left(x,t\right)\Phi_{j}\left(x\right)dx=0 \end{array}$$

In matrix notation, we define,

(36) $$\left[C\right]\;\left\{\frac{da(\tau )}{d\tau }\right\}+\left[K\right]\;\left\{a\left(\tau \right)\right\}=\left\{F\right\}$$

where,

$$\begin{array}{cc} & C_{ij}=\int_{0}^{1}\Phi_{j}\left(x\right)\Phi_{i}\left(x\right)dx\\ & K_{ij}=-\int_{0}^{1}\left(\frac{\partial^{1.8}}{\partial x^{1.8}}\Phi_{j}\left(x\right)\right)\Phi_{i}\left(x\right)q\left(x\right)dx\\ & F_{i}=\int_{0}^{1}s\left(x,\tau \right)\Phi_{i}\left(x\right)dx \end{array}$$

Here, $\Phi_{1}\left(x\right)=x^{2}-x$, $\Phi_{2}\left(x\right)=\frac{x}{2}-\frac{3x^{2}}{2}+x^{3}$. Hence, we obtain,

$\left[C\right]=\left(\begin{array}{cc} 0.0333 & 0\\ 0 & 0.0012 \end{array}\right)$$\left[K\right]=\left(\begin{array}{cc} 0.0833 & 0.0250\\ 0.0150 & 0.0115 \end{array}\right)$$\left\{F\right\}=\left(\begin{array}{c} \frac{-e^{-t}}{20}\\ \frac{-e^{-t}}{56} \end{array}\right)$ Substituting [C],[K]and{F} into Eq. (36), we get

$$\begin{array}{cc} & a_{1}\left(t\right)=-0.09e^{-10.8t}\left(1.55e^{9.83t}-1.34e^{-12}\right)-0.661e^{-1.37t}\left(0.546e^{0.366t}-1.61e^{-12}\right)\\ & a_{2}\left(t\right)=1.0e^{-1.37t}\left(0.546e^{0.366t}-1.61e^{-12}\right)-e^{-10.8t}\left(1.55e^{9.83t}-1.34e^{-12}\right) \end{array}$$

Thus, the approximate solution is

$$\begin{array}{cc} & \tilde{u}\left(x,t\right)=\left(0.09e^{-10.8t}\left(1.55e^{9.83t}-1.34e^{-12}\right)+0.661e^{-1.37t}\left(0.546e^{0.366t}-1.61e^{-12}\right)\right)\left(-x^{2}+x\right)\\ & \;-\left(e^{-10.8t}\left(1.55e^{9.83t}-1.34e^{-12}\right)-e^{-1.37t}\left(0.546e^{0.366t}-1.61e^{-12}\right)\right)\left(x^{3}-1.5x^{2}+0.5x\right). \end{array}$$

**Fig. 1:**
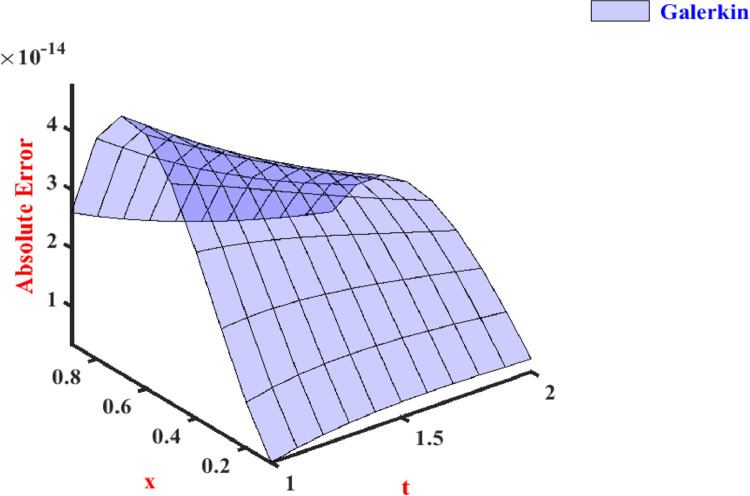
Absolute errors for Problem-1.

In [46], shifted Chebyshev polynomials of the second kind were used to solve the problem. Abbas and Mehdi solved the space fractional diffusion equation using a numerical technique based on the shifted Legendre tau method in [30]. In [31], the fractional diffusion equation is reduced to a system of ordinary differential equations by using the properties of Chebyshev polynomials. In [32], the collocation method using the fourth kind of Chebyshev polynomial and compact finite difference method has been used. Now we compute approximate values and absolute errors using two parameters which are displayed in Table 1, Table 2, and Table 3 at different values of time. In these tables, we compare our obtained results with the existing values [30], [31], [32], [46]. These tables show that the Galerkin method provides us with better accuracy for various values of x, and we may observe that the Galerkin approach has outperformed the existing methods described in [30], [31], [32], [46]. The present view of Fig. 1 shows the absolute errors of our proposed method for space fractional PDE.

**Table 1:** Absolute errors for Problem-1 at t=1.

x	with N=3 in [46]	with N=3 in [32]	with N=7 in [31] reported in [32], [46]	Present method with N=2
0.10	5.46$\times 10^{-06}$	3.17$\times 10^{-09}$	4.26$\times 10^{-5}$	3.52$\times 10^{-15}$
0.20	8.51$\times 10^{-06}$	5.85$\times 10^{-09}$	5.39$\times 10^{-5}$	1.13$\times 10^{-14}$
0.30	9.60$\times 10^{-06}$	7.97$\times 10^{-09}$	6.12$\times 10^{-5}$	2.14$\times 10^{-14}$
0.40	9.18$\times 10^{-06}$	9.44$\times 10^{-09}$	6.48$\times 10^{-5}$	3.19$\times 10^{-14}$
0.50	7.69$\times 10^{-06}$	1.02$\times 10^{-08}$	6.45$\times 10^{-5}$	4.11$\times 10^{-14}$
0.60	5.60$\times 10^{-06}$	1.01$\times 10^{-08}$	5.98$\times 10^{-5}$	4.60$\times 10^{-14}$
0.70	3.33$\times 10^{-06}$	9.12$\times 10^{-09}$	5.23$\times 10^{-5}$	4.77$\times 10^{-14}$
0.80	1.34$\times 10^{-06}$	7.17$\times 10^{-09}$	4.48$\times 10^{-5}$	4.13$\times 10^{-14}$
0.90	8.39$\times 10^{-08}$	4.16$\times 10^{-09}$	3.91$\times 10^{-5}$	2.61$\times 10^{-14}$

**Table 2:** Absolute errors for Problem-1 at t=2.

x	with N=3 in [46]	with N=5 in [30]	with N=3 in [31]	with N=7 in [32]	Present method with N=2
0.10	3.33$\times 10^{-06}$	4.47$\times 10^{-06}$	4.18$\times 10^{-06}$	1.28$\times 10^{-08}$	5.71$\times 10^{-15}$
0.20	5.65$\times 10^{-06}$	2.78$\times 10^{-07}$	5.45$\times 10^{-06}$	2.05$\times 10^{-08}$	1.13$\times 10^{-14}$
0.30	7.05$\times 10^{-06}$	5.81$\times 10^{-06}$	6.18$\times 10^{-06}$	2.40$\times 10^{-08}$	1.62$\times 10^{-14}$
0.40	7.64$\times 10^{-06}$	1.02$\times 10^{-05}$	6.49$\times 10^{-06}$	2.40$\times 10^{-08}$	2.02$\times 10^{-14}$
0.50	7.52$\times 10^{-06}$	1.17$\times 10^{-05}$	6.40$\times 10^{-06}$	2.15$\times 10^{-08}$	2.28$\times 10^{-14}$
0.60	6.80$\times 10^{-06}$	1.08$\times 10^{-05}$	5.95$\times 10^{-06}$	1.72$\times 10^{-08}$	2.35$\times 10^{-14}$
0.70	5.59$\times 10^{-06}$	8.54$\times 10^{-06}$	5.32$\times 10^{-06}$	1.21$\times 10^{-08}$	2.20$\times 10^{-14}$
0.80	3.98$\times 10^{-06}$	6.06$\times 10^{-06}$	4.60$\times 10^{-06}$	6.93$\times 10^{-09}$	1.79$\times 10^{-14}$
0.90	2.08$\times 10^{-06}$	3.67$\times 10^{-06}$	3.79$\times 10^{-06}$	2.62$\times 10^{-09}$	1.07$\times 10^{-14}$

**Table 3:** Absolute errors for Problem-1 at t=10.

x	with N=7 in [32]	with N=7 in [46]	Present method with N=2
0.20	2.28$\times 10^{-09}$	2.34$\times 10^{-08}$	8.88$\times 10^{-18}$
0.40	4.21$\times 10^{-09}$	4.78$\times 10^{-09}$	1.28$\times 10^{-17}$
0.60	1.15$\times 10^{-09}$	7.39$\times 10^{-09}$	1.24$\times 10^{-17}$
0.80	2.71$\times 10^{-10}$	2.84$\times 10^{-08}$	7.92$\times 10^{-18}$

**Problem-2:** We are considering the Eq. (4) with time and space fractional diffusion equation as [45]:

(37) $$\frac{\partial^{\alpha }u}{\partial t^{\alpha }}=\frac{\partial^{\beta }u}{\partial x^{\beta }}+f\left(x,t\right)$$

for 0<α<1 and 1<β<2, and

$$f\left(x,t\right)=\frac{\Gamma (3+\alpha )}{2}x^{2}\left(x-1\right)t^{2}-2x^{\left(2-\beta \right)}t^{\left(2+\alpha \right)}\left[\frac{3x}{\Gamma (4-\beta )}-\frac{1}{\Gamma (3-\beta )}\right],$$

with the boundary and initial conditions: u(0,t)=u(1,t)=0 and u(x,0)=0. The exact solution for this problem is, $u\left(x,t\right)=x^{2}\left(x-1\right)t^{\left(2+\alpha \right)}$

Since both time and space contain fractional derivatives we have fixed the values of derivative order such as α=0.2andβ=1.6 for this problem. To start with the solution to this problem, as basis functions, we have chosen only two modified Bernoulli polynomials. Due to the presence of fractional time derivative, we have solved this problem using the Galerkin- Finite Difference method. At first, we choose an approximate solution for this problem in the following form:

(38) $$\tilde{u}\left(x,\tau_{n}\right)=\Phi_{0}\left(x,\tau_{n}\right)+\sum_{i=1}^{2}a_{i}\left(\tau_{n}\right)\Phi_{i}\left(x\right)$$

where, $\phi_{0}\left(x,\tau \right)$ represents non-homogeneous part of our boundary condition and $\phi_{0}\left(x,\tau_{n}\right)$ displays it’s value at any specified time. Since we already have homogeneous boundary conditions so we can have, $\phi_{0}\left(x,\tau_{n}\right)=0.$ So, our new approximate solution becomes,

(39) $$\tilde{u}\left(x,\tau_{n}\right)=\sum_{i=1}^{2}a_{i}\left(\tau_{n}\right)\Phi_{i}\left(x\right)$$

To approximate the solution of Eq. (37) using the Galerkin method we solve the following integration, and obtain a set of linear equations,

(40) $$\int_{0}^{1}R\left(x,\tau_{n+1}\right)\Phi_{j}\left(x\right)dx=0$$

Putting the residual function into Eq. (40) we get

$$\int_{0}^{1}(V\left[\tilde{u}\left(x,\tau_{n+1}\right)-\tilde{u}\left(x,\tau_{n}\right)+\sum_{l=1}^{n}\left(\tilde{u}\left(x,\tau_{n+1-l}\right)-\tilde{u}\left(x,\tau_{n-l}\right)\right)P_{l}\right]-\left[\frac{\partial^{\beta }\tilde{u}\left(x,\tau_{n+1}\right)}{\partial x^{\beta }}\right]-f\left(x,\tau_{n+1}\right))\Phi_{j}\left(x\right)dx=0$$

Using Eq. (39) in the above equation we can obtain,

$$\begin{array}{cc} & V[\sum_{i=1}^{2}a_{i}\left(\tau_{n+1}\right)\int_{0}^{1}\left(\Phi_{i}\left(x\right)\Phi_{j}\left(x\right)\right)dx-\sum_{i=1}^{2}a_{i}\left(\tau_{n}\right)\int_{0}^{1}\left(\Phi_{i}\left(x\right)\Phi_{j}\left(x\right)\right)dx\\ & +\sum_{l=1}^{n}(\sum_{i=1}^{2}a_{i}\left(\tau_{n+1-l}\right)\int_{0}^{1}\left(\Phi_{i}\left(x\right)\Phi_{j}\left(x\right)\right)dx-\sum_{i=1}^{2}a_{i}\left(\tau_{n-l}\right)\int_{0}^{1}\left(\Phi_{i}\left(x\right)\Phi_{j}\left(x\right)\right)dx)P_{l}]\\ & -\sum_{i=1}^{2}a_{i}\left(\tau_{n+1}\right)\int_{0}^{1}[\frac{\partial^{\beta }\left(\Phi_{i}\left(x\right)\right)}{\partial x^{\beta }}]\Phi_{j}\left(x\right)dx-\int_{0}^{1}\left(f\left(x,\tau_{n+1}\right)\Phi_{j}\left(x\right)\right)dx=0 \end{array}$$

For, n=0 we get two linear equations as following:

(41) $$\begin{array}{cc} & V[\int_{0}^{1}\left(\Phi_{1}^{2}\left(x\right)\right)dx\left(a_{1}\left(\tau_{1}\right)-a_{1}\left(\tau_{0}\right)\right)+\int_{0}^{1}\left(\Phi_{1}\left(x\right)\Phi_{2}\left(x\right)\right)dx\left(a_{2}\left(\tau_{1}\right)-a_{2}\left(\tau_{0}\right)\right)]\\ & =\int_{0}^{1}\left(\Phi_{1}^{2}\left(x\right)\right)dx\left(a_{1}\left(\tau_{1}\right)\right)+\int_{0}^{1}\left(\Phi_{1}\left(x\right)\Phi_{2}\left(x\right)\right)dx\left(a_{2}\left(\tau_{1}\right)\right)+\int_{0}^{1}\left(f\left(x,\tau_{1}\right)\Phi_{1}\left(x\right)\right)dx \end{array}$$

(42) $$\begin{array}{cc} & V[\int_{0}^{1}\left(\Phi_{1}\left(x\right)\Phi_{2}\left(x\right)\right)dx\left(a_{1}\left(\tau_{1}\right)-a_{1}\left(\tau_{0}\right)\right)+\int_{0}^{1}\left(\Phi_{2}^{2}\left(x\right)\right)dx\left(a_{2}\left(\tau_{1}\right)-a_{2}\left(\tau_{0}\right)\right)]\\ & =\int_{0}^{1}\left(\Phi_{1}\left(x\right)\Phi_{2}\left(x\right)\right)dx\left(a_{1}\left(\tau_{1}\right)\right)+\int_{0}^{1}\left(\Phi_{2}^{2}\left(x\right)\right)dx\left(a_{2}\left(\tau_{1}\right)\right)+\int_{0}^{1}\left(f\left(x,\tau_{1}\right)\Phi_{2}\left(x\right)\right)dx \end{array}$$

Now we substitute the values of unknown parameters $\left(a_{1},a_{2}\right)$ at initial time state $\left(\tau_{0}=0\right)$ from the given initial condition via following the steps discussed below:

(43) $$\int_{0}^{1}(\tilde{u}\left(x,0\right)-u\left(x,0\right))\Phi_{j}\left(x\right)dx=0$$

Applying the approximate value from Eq. (39) into (43) we get,

(44) $$\int_{0}^{1}(\sum_{i=1}^{2}a_{i}\left(0\right)\Phi_{i}\left(x\right)-0)\Phi_{j}\left(x\right)dx=0$$

From Eq. (44) we get a set of linear equations which provide us the values of unknowns. Repeating the process for increasing time steps we obtain our desired result. For this problem, we take the basis functions as:

$\Phi_{1}\left(x\right)=x^{2}-x$ and $\Phi_{2}\left(x\right)=\frac{x}{2}-\frac{3x^{2}}{2}+x^{3}$, and hence we get, $\left[G\right]=\left(\begin{array}{cc} 0.0333 & 0\\ 0 & 0.0012 \end{array}\right)$, V=1.7017, $\left[K\right]=\left(\begin{array}{cc} -0.1421 & 0.0244\\ -0.0244 & -0.0171 \end{array}\right)$ and $\left\{N\right\}=\left(\begin{array}{c} 0.0202t^{2}+0.04664t^{\frac{11}{5}}\\ 0.001443t^{2}+0.0293t^{\frac{11}{5}} \end{array}\right).$

The solution of this problem is not similar to Eq. (37) due to the discretization of the time-fractional derivative. So, our approximated solution is represented at t=1 as, $u\left(x,1\right)=1.01x^{3}-1.02x^{2}+0.0127x$ and at t=0.5 as, $u\left(x,0.5\right)=0.219x^{3}-0.222x^{2}+0.00323x$

Now, we compute absolute error and order of convergence to compare the results with the existing literature [47], which are shown in Tables 4 and 5 for different values of *α* and *β*.

**Table 4:** Error analysis for Problem-2 for α=0.4 and β=1.75.

δt	For $\delta \;x=\frac{1}{1000}$ in [45]		Present method for $\delta \;x=\frac{1}{10}$	
	Absolute Error	Convergence order C(δt)	Absolute Error	Convergence order C(δt)
$\frac{1}{20}$	8.535166$\times 10^{-05}$	-	4.521834$\times 10^{-03}$	-
$\frac{1}{40}$	2.901776$\times 10^{-05}$	1.5565	3.495112$\times 10^{-03}$	1.2938
$\frac{1}{80}$	9.718379$\times 10^{-06}$	1.5781	2.553417$\times 10^{-03}$	1.3688
$\frac{1}{160}$	3.176376$\times 10^{-06}$	1.6133	1.804433$\times 10^{-03}$	1.4151

**Table 5:** Error analysis for Problem-2 for α=0.5 and β=1.88.

δt	For $\delta \;x=\frac{1}{1000}$ in [45]		Present method for $\delta \;x=\frac{1}{10}$	
	Absolute Error	Convergence order C(δt)	Absolute Error	Convergence order C(δt)
$\frac{1}{20}$	1.326761$\times 10^{-04}$	-	5.600296$\times 10^{-03}$	-
$\frac{1}{40}$	4.803713$\times 10^{-05}$	1.4657	4.587591$\times 10^{-03}$	1.2207
$\frac{1}{80}$	1.722465$\times 10^{-05}$	1.4797	3.550801$\times 10^{-03}$	1.2919
$\frac{1}{160}$	6.114506$\times 10^{-06}$	1.4942	2.660682$\times 10^{-03}$	1.3345

**Fig. 2:**
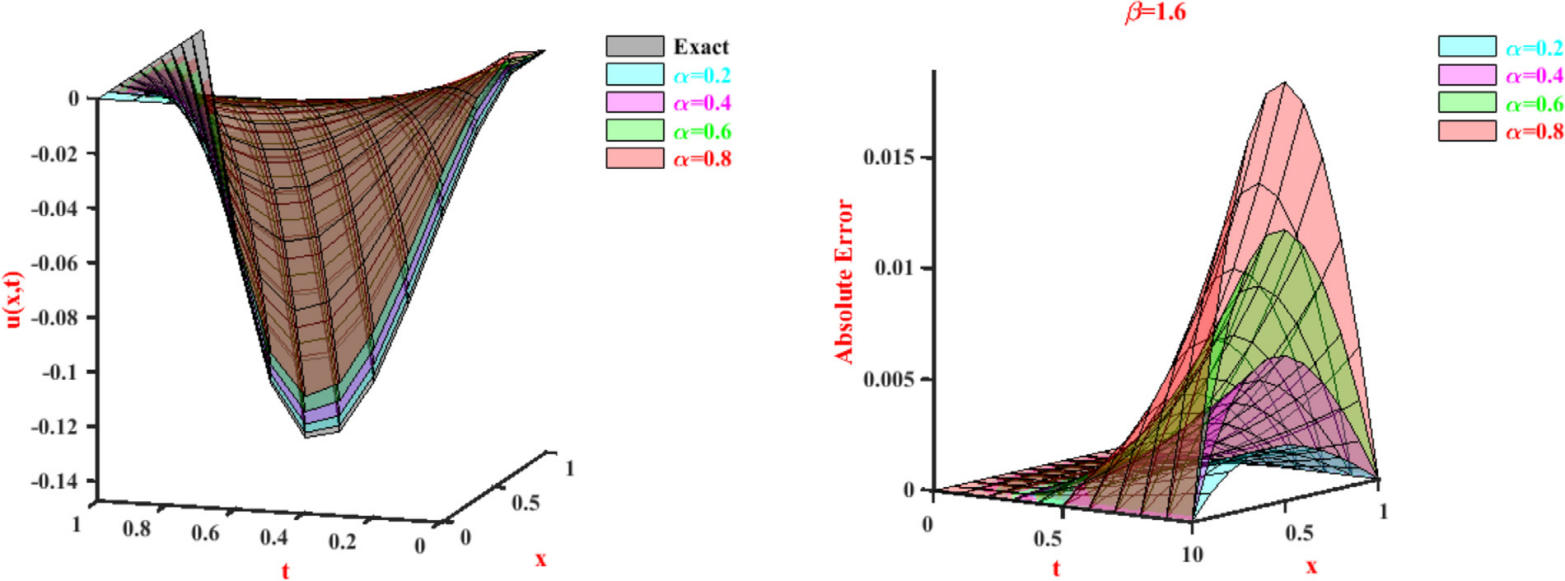
Values and absolute errors for Problem-2 with β=1.6.

**Fig. 3:**
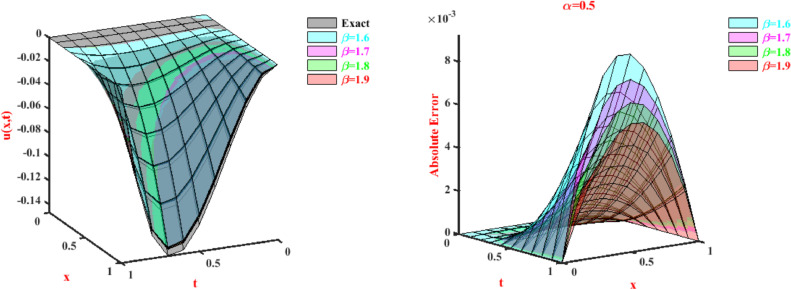
Values and absolute errors for Problem-2 with α=0.5.

To analyze the convergence order we have considered maximum absolute error for different time steps and displayed them in Table 6, and we observe that the order of convergence is ensured. The tabular representation of absolute errors for Problem-2 is shown in Table 7 and Table 8. We generate Table 7 and Table 8 by changing one of the parameters (α, β) and keeping fixed the other. According to the tables, for fixed β=1.6 we get better values of u(x,t) at α=0.2. and for fixed α=0.5 we get good approximation values of u(x,t) at β=1.8. The 3-D views for approximate values and the corresponding absolute errors are shown in Fig. 2 and Fig. 3 for fixed β and α, respectively.

**Table 6:** Error analysis for Problem-2 at t=0.5.

δt	$L_{\infty }$ norm	Convergence order C(δt)
1/10	6.1400$\times 10^{-04}$	-
1/20	4.6870$\times 10^{-04}$	1.31
1/40	3.2487$\times 10^{-04}$	1.44

**Table 7:** Absolute errors for Problem-2 with N=2 at t=0.5 and fixed β.

x	β=1.6			
	α=0.2	α=0.4	α=0.6	α=0.8
0.1	2.01108$\;\times \;10^{-04}$	5.50913$\;\times \;10^{-04}$	1.02393$\;\times \;10^{-03}$	1.42967$\;\times \;10^{-03}$
0.2	3.41886$\;\times \;10^{-04}$	9.43631$\;\times \;10^{-04}$	1.77906$\;\times \;10^{-03}$	2.55309$\;\times \;10^{-03}$
0.3	4.28197$\;\times \;10^{-04}$	1.19157$\;\times \;10^{-03}$	2.28086$\;\times \;10^{-03}$	3.36595$\;\times \;10^{-03}$
0.4	4.65908$\;\times \;10^{-04}$	1.30814$\;\times \;10^{-03}$	2.54480$\;\times \;10^{-03}$	3.86397$\;\times \;10^{-03}$
0.5	4.60884$\;\times \;10^{-04}$	1.30676$\;\times \;10^{-03}$	2.58636$\;\times \;10^{-03}$	4.04286$\;\times \;10^{-03}$
0.6	4.18988$\;\times \;10^{-04}$	1.20083$\;\times \;10^{-03}$	2.42101$\;\times \;10^{-03}$	3.89832$\;\times \;10^{-03}$
0.7	3.46087$\;\times \;10^{-04}$	1.00378$\;\times \;10^{-03}$	2.06422$\;\times \;10^{-03}$	3.42605$\;\times \;10^{-03}$
0.8	2.48045$\;\times \;10^{-04}$	7.29017$\;\times \;10^{-04}$	1.53148$\;\times \;10^{-03}$	2.62177$\;\times \;10^{-03}$
0.9	1.30728$\;\times \;10^{-04}$	3.89952$\;\times \;10^{-04}$	8.38245$\;\times \;10^{-04}$	1.48119$\;\times \;10^{-03}$

**Table 8:** Absolute errors for Problem-2 with N=2 at t=0.5 and fixed α.

x	α=0.5			
	β=1.2	β=1.4	β=1.6	β=1.8
0.1	1.71993$\;\times \;10^{-03}$	1.18037$\;\times \;10^{-03}$	7.79530$\;\times \;10^{-04}$	4.94728$\;\times \;10^{-04}$
0.2	2.76587$\;\times \;10^{-03}$	1.95704$\;\times \;10^{-03}$	1.34320$\;\times \;10^{-03}$	8.92565$\;\times \;10^{-04}$
0.3	3.24723$\;\times \;10^{-03}$	2.38302$\;\times \;10^{-03}$	1.70700$\;\times \;10^{-03}$	1.18862$\;\times \;10^{-03}$
0.4	3.27344$\;\times \;10^{-03}$	2.51134$\;\times \;10^{-03}$	1.88691$\;\times \;10^{-03}$	1.37800$\;\times \;10^{-03}$
0.5	2.95391$\;\times \;10^{-03}$	2.39503$\;\times \;10^{-03}$	1.89893$\;\times \;10^{-03}$	1.45580$\;\times \;10^{-03}$
0.6	2.39807$\;\times \;10^{-03}$	2.08712$\;\times \;10^{-03}$	1.75903$\;\times \;10^{-03}$	1.41714$\;\times \;10^{-03}$
0.7	1.71534$\;\times \;10^{-03}$	1.64064$\;\times \;10^{-03}$	1.48320$\;\times \;10^{-03}$	1.25713$\;\times \;10^{-03}$
0.8	1.01514$\;\times \;10^{-03}$	1.10860$\;\times \;10^{-03}$	1.08742$\;\times \;10^{-03}$	9.70862$\;\times \;10^{-04}$
0.9	4.06885$\;\times \;10^{-04}$	5.44049$\;\times \;10^{-04}$	5.87697$\;\times \;10^{-04}$	5.53450$\;\times \;10^{-04}$

#### Application in Financial Mathematics

Here the famous Black-Scholes model is taken into consideration. This model equation is a modified version of parabolic PDE or diffusion equation. At first, this equation is transformed into a parabolic type equation using appropriate transformations and then into a fractional order parabolic type equation, which can be easily computed numerically, details are available in [[47], [48], [49], [50], [51]]. To approximate option price, we analyze the following Black-Scholes Partial Differential equation:

(45) $$\frac{\partial \chi }{\partial t}+rS\frac{\partial \chi }{\partial S}+\frac{1}{2}\sigma^{2}S^{2}\frac{\partial^{2}\chi }{\partial S^{2}}-r\chi =0,\;0<S<\infty ,\;t\in \left[0,T\right]$$

subject to the terminal and boundary conditions for European call and put options:

χ(S,t)=max(S−K,0), when t=T;χ(S,t)=0, when $S\to 0;\;\chi \left(S,t\right)=S-Ke^{-r(T-t)}$, when S→∞ and χ(S,t)=max(K−S,0), when $t=T;\;\chi \left(S,t\right)=Ke^{-r(T-t)}$; when S→0,χ(S,t)=0, when S→∞ respectively. Throughout this paper, we use the notations: χ=χ(S,t)= the option price, S= stock price, K= strike price, T= maturity time, r= interest rate, t= time in years, and σ= volatility.

At first we apply the modifications [52]: $y=ln(\frac{S}{K})$, $\tau =\frac{\sigma^{2}}{2}\left(T-t\right)$ where, a≤y≤b and 0≤τ≤T into Eq. (45).

##### Limit change

We define $a=\ln(\frac{min(S)}{K})$ and $b=\ln(\frac{max(S)}{K})$ as our new boundary value, and y=a+(b−a)x we get,

(46) $$\frac{\partial u}{\partial \tau }=\frac{1}{{\left(b-a\right)}^{2}}\frac{\partial^{2}u}{\partial x^{2}}$$

where, 0≤x≤1 and $0\leq \tau \leq \frac{\sigma^{2}}{2}T$.

Now the Black-Scholes PDE defined in Eq. (45) becomes to a fractional order in the space variable which is similar to [47]:

(47) $$\frac{\partial u}{\partial \tau }=\frac{1}{{\left(b-a\right)}^{2}}\frac{\partial^{\beta }u}{\partial x^{\beta }}$$

On the other hand, we may consider the above equation in both space and time fractional derivatives as following [45]:

(48) $$\frac{\partial^{\alpha }u}{\partial \tau^{\alpha }}=\frac{1}{{\left(b-a\right)}^{2}}\frac{\partial^{\beta }u}{\partial x^{\beta }}$$

Now we state the corresponding boundary and initial conditions for European options:

Put option:

Boundary Condition : $u\left(0,\tau \right)=e^{-pa+p^{2}\tau }$ and u(1,τ)=0 for τ>0

Initial Condition : $u\left(x,0\right)=e^{-p(a+(b-a)x)}max\left(1-e^{a+(b-a)x},0\right)$; where, 0<x<1.

Call option:

Boundary Condition : $u\left(1,\tau \right)=e^{(1-p)b-q\tau }-e^{-pb+p^{2}\tau }$ and u(0,τ)=0 for τ>0

Initial Condition : $u\left(x,0\right)=e^{-p(a+(b-a)x)}max\left(e^{a+(b-a)x}-1,0\right)$; where, 0<x<1.

**Fig. 4:**
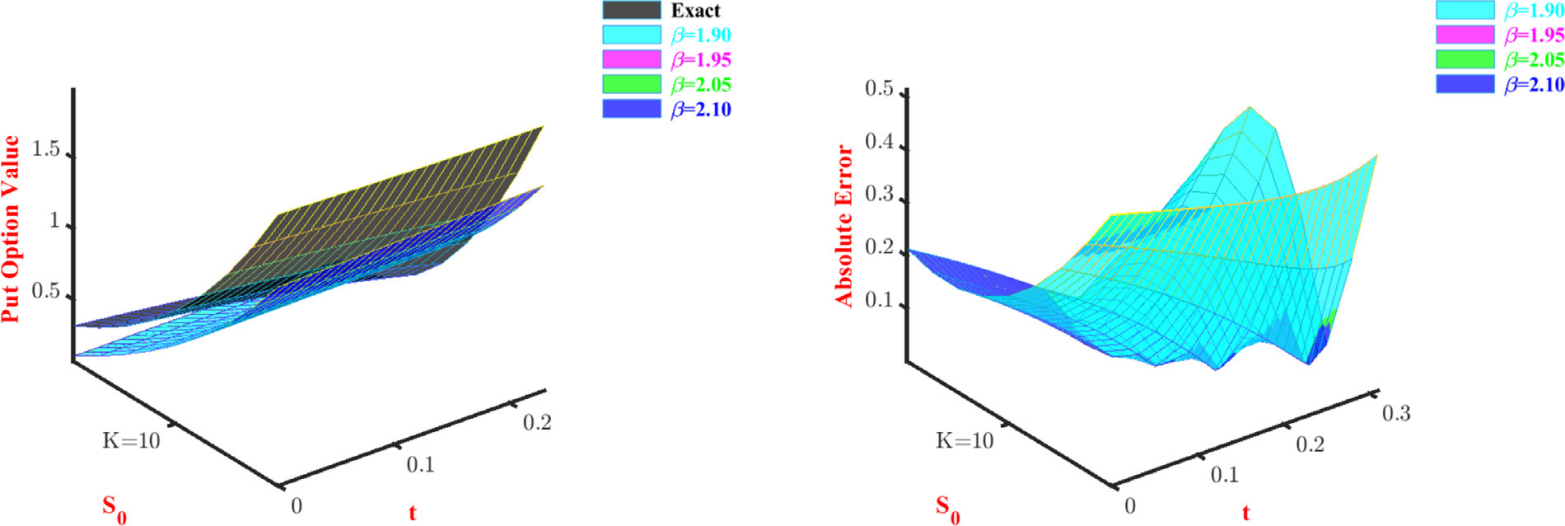
Prices and absolute errors to approximate Put option values for Problem-3.

**Fig. 5:**
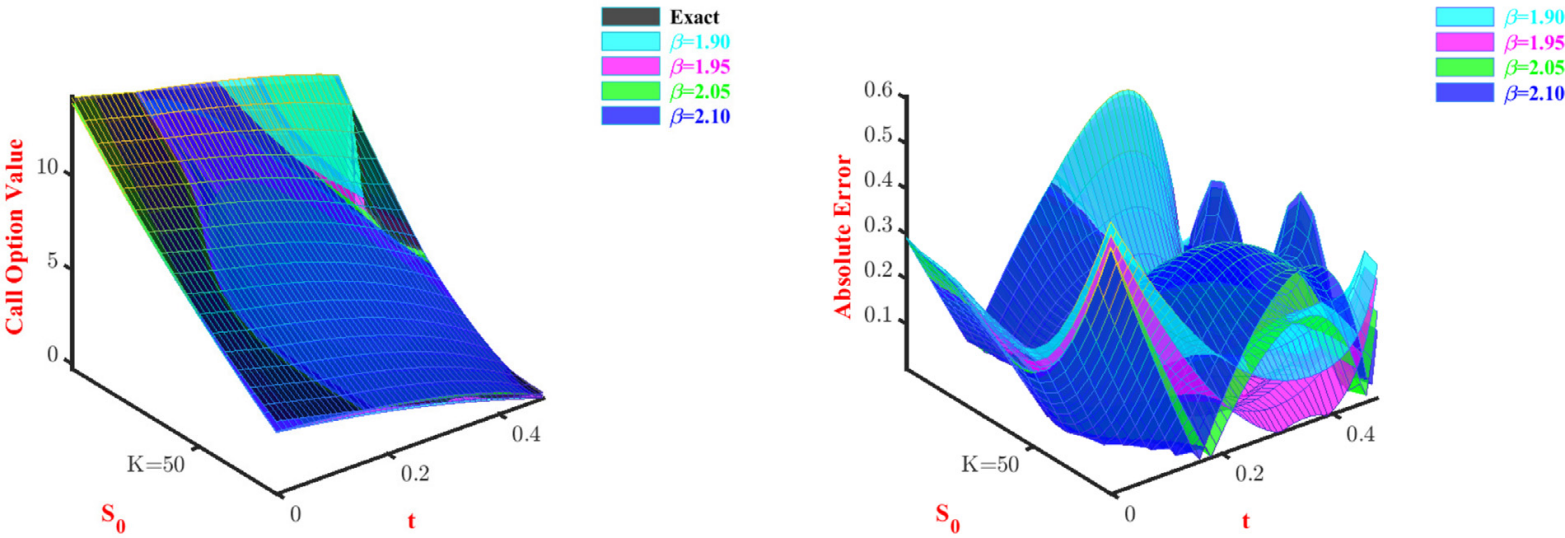
Prices and absolute errors to approximate Call option values for Problem-3.

**Problem-3:**Eq. (47) is solved using the Galerkin method, discussed for space fractional PDE. Here we also consider two parameters and two modified Bernoulli polynomials as basis functions. Table 9 and Table 10 represent approximate call option and put option values, respectively. As we can observe from Table 9, with an increase in the stock price approximate values converge towards the exact option price when we increase the values of β. On the other hand, Table 10 shows that among all the values of β, the method has performed better at β=1.9, and almost for all values of stock price the approximate price converges towards the exact option price. 3D visual representation of the price and absolute error corresponding to this problem is shown through Fig. 4 and Fig. 5 for the put option and call option, respectively. The current view of Fig. 4 and Fig. 5 display through our proposed method discussed for space fractional PDE, provides poor approximations in some cases for β=1.90 and β=2.10 but gives good approximations for all values of β all the time.

**Table 9:** Approximate Call option value for Problem-3 with N=2.

$S_{0}$	Exact	β=1.9	β=1.95	β=2.05	β=2.1
46.0	4.5167	3.9990	3.9943	3.9336	3.8797
47.0	5.0412	4.5953	4.5942	4.5410	4.4902
48.0	5.5956	5.2017	5.2046	5.1604	5.1141
49.0	6.1790	5.8191	5.8261	5.7921	5.7511
50.0	6.7902	6.4482	6.4591	6.4358	6.4007
51.0	7.4282	7.0893	7.1040	7.0916	7.0628
52.0	8.0919	7.7430	7.7612	7.7593	7.7368
53.0	8.7801	8.4095	8.4307	8.4388	8.4226
54.0	9.4914	9.0891	9.1128	9.1299	9.1197
55.0	10.2247	9.7820	9.8076	9.8325	9.8277

**Table 10:** Approximate Put option value for Problem-3 with N=2.

$S_{0}$	Exact	β=1.9	β=1.95	β=2.05	β=2.1
6.0	3.6977	3.9089	3.9071	3.9033	3.9014
7.0	2.7809	3.2014	3.5469	3.5434	3.5417
8.0	1.9806	2.5417	3.1998	3.1966	3.1950
9.0	1.3377	1.9224	2.8646	2.8617	2.8602
10.0	0.8610	1.3377	2.5404	2.5377	2.5364
11.0	0.5318	0.7831	2.2262	2.2238	2.2227
12.0	0.3174	0.2551	1.9213	1.9193	1.9182

**Problem-4:**Eq. (48) holds for both the options (call and put) but boundary conditions and initial condition are different for call option and put option. In this case, the initial and boundary conditions are similar to the conditions stated in Problem-3. Since this problem falls in the category of space and time fractional parabolic partial differential equation we are ready to solve by the method described for time-space fractional PDE which is similar to Problem 2. Thus we solve this problem as following problem 2 and we prepare the Table 11 to Table 16 subsequently.

**Table 11:** Approximate Call option value for Problem-4 with N=2 and fixed α.

$S_{0}$	Exact	α=0.5			
		β=1.2	β=1.4	β=1.6	β=1.8
46.0	4.5167	9.9961	7.8169	5.7006	4.0686
47.0	5.0412	11.5734	9.3427	7.0312	5.1813
48.0	5.5956	12.5320	10.4757	8.1472	6.1980
49.0	6.1790	13.0009	11.2892	9.0839	7.1317
50.0	6.7902	13.0943	11.8488	9.8728	7.9940
51.0	7.4282	12.9136	12.2124	10.5418	8.7952
52.0	8.0919	12.5487	12.4315	11.1159	9.5447
53.0	8.7801	12.0793	12.5521	11.6173	10.2509
54.0	9.4914	11.5760	12.6150	12.0660	10.9212
55.0	10.2247	11.1015	12.6563	12.4796	11.5623

Table 11 and Table 12 represent the approximate call option values for fixed α and β, respectively. Our observation from Table 11 is that at β=1.8, our proposed method has performed better for almost all the values of stock price, and approximate values converge towards the exact option price. And when we fixed the value of α we see from Table 12 that for fixed β=1.6, all the values of α provide almost similar results. The approximate values with the exact option price in the case of put options are shown in Table 13 and Table 14. Table 13 shows us that with a fixed value of α, when we increase stock price the approximate values of increasing β converge towards the exact option price. From, Table 14 we see that for a fixed value of β with an increase in stock price, we get a better approximation with a decrease in the values of α before strike price. But for the stock prices higher than the strike price, there is a linear relationship between stock price and approximate values of different α.

**Table 12:** Approximate Call option value for Problem-4 with N=2 and fixed β.

$S_{0}$	Exact	β=1.6			
		α=0.2	α=0.4	α=0.6	α=0.8
46.0	4.5167	5.7160	5.7028	5.7017	5.7133
47.0	5.0412	7.0500	7.0340	7.0321	7.0451
48.0	5.5956	8.1686	8.1506	8.1479	8.1612
49.0	6.1790	9.1073	9.0879	9.0842	9.0973
50.0	6.7902	9.8976	9.8772	9.8726	9.8849
51.0	7.4282	10.5674	10.5466	10.5411	10.5521
52.0	8.0919	11.1418	11.1210	11.1147	11.1242
53.0	8.7801	11.6431	11.6226	11.6156	11.6234
54.0	9.4914	12.0912	12.0714	12.0639	12.0699
55.0	10.2247	12.5038	12.4850	12.4771	12.4813

**Table 13:** Approximate Put option value for Problem-4 with N=2 and fixed α.

$S_{0}$	Exact	α=0.5			
		β=1.2	β=1.4	β=1.6	β=1.8
6.0	3.6977	3.3489	3.3247	3.3154	3.3118
7.0	2.7809	2.7187	2.7018	2.6980	2.6979
8.0	1.9806	2.1499	2.1386	2.1383	2.1402
9.0	1.3377	1.6307	1.6236	1.6253	1.6281
10.0	0.8610	1.1523	1.1483	1.1508	1.1537
11.0	0.5318	0.7085	0.7066	0.7088	0.7111
12.0	0.3174	0.2940	0.2934	0.2946	0.2958

**Table 14:** Approximate Put option value for Problem-4 with N=2 and fixed β.

$S_{0}$	Exact	β=1.6			
		α=0.2	α=0.4	α=0.6	α=0.8
6.0	3.6977	3.1086	3.2368	3.3980	3.5553
7.0	2.7809	2.5265	2.6321	2.7682	2.9038
8.0	1.9806	2.0004	2.0848	2.1960	2.3086
9.0	1.3377	1.5192	1.5838	1.6705	1.7595
10.0	0.8610	1.0750	1.1209	1.1836	1.2489
11.0	0.5318	0.6618	0.6901	0.7295	0.7709
12.0	0.3174	0.2750	0.2868	0.3034	0.3211

**Fig. 6:**
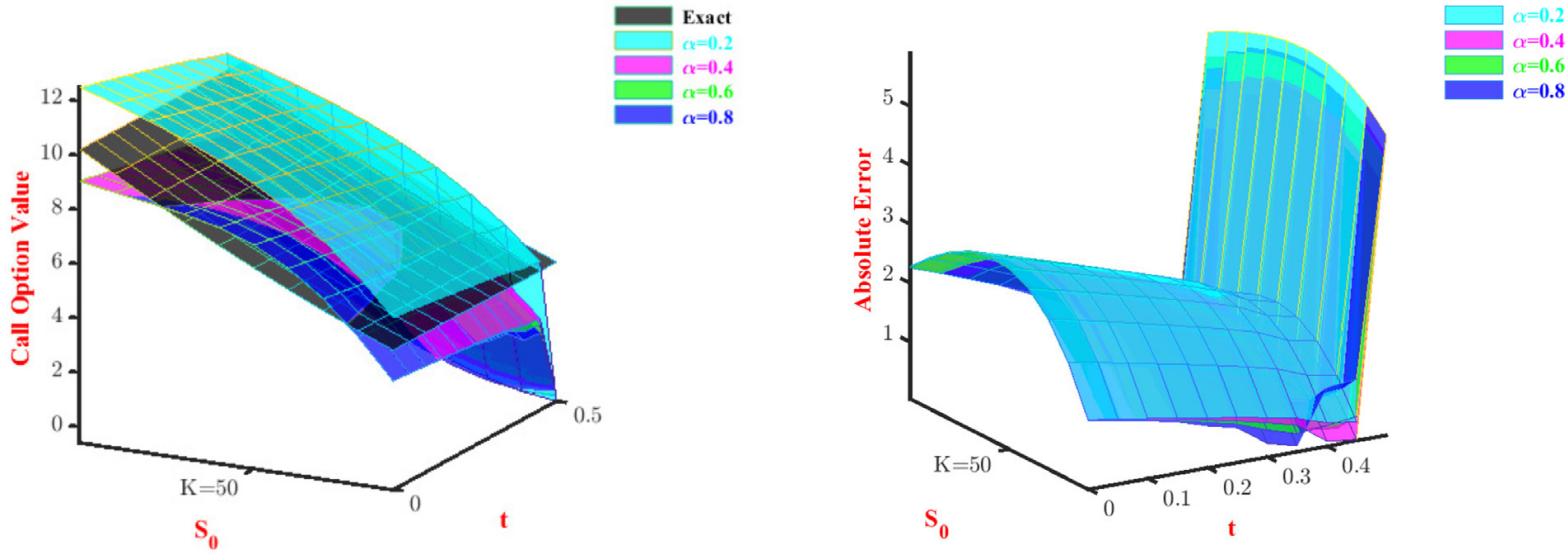
Prices and absolute errors to approximate Call option values for Problem-4 with β=1.6.

**Fig. 7:**
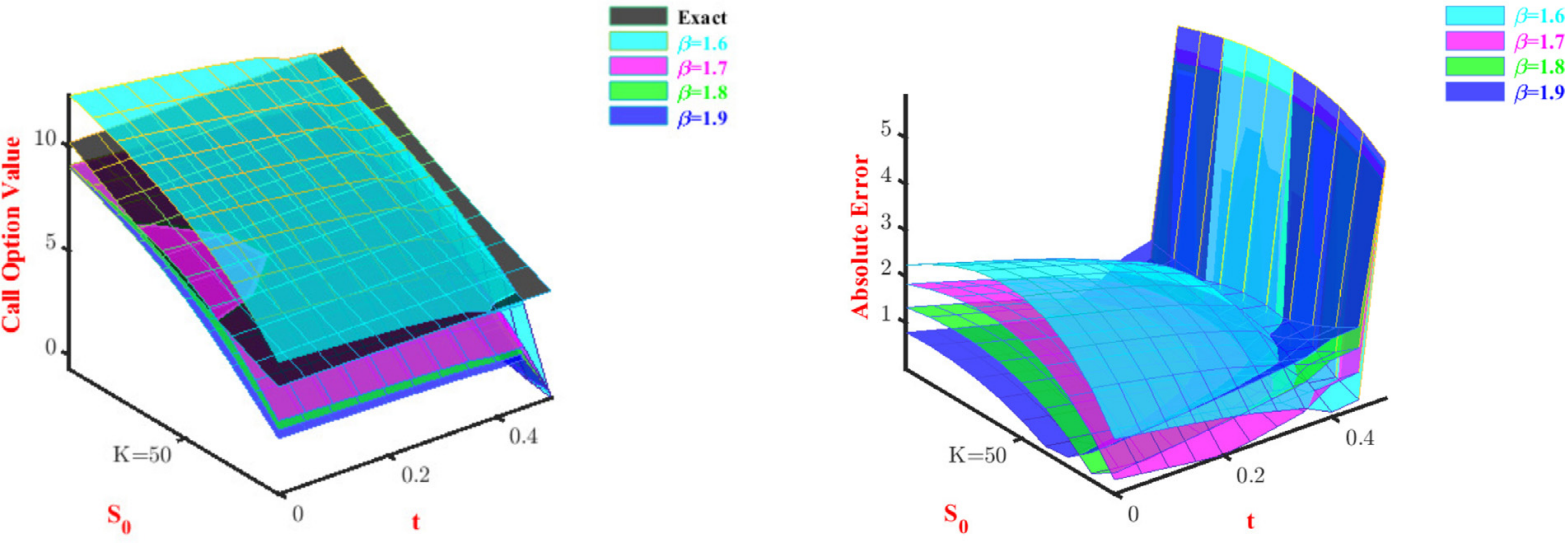
Prices and absolute errors to approximate Call option values for Problem-4 with α=0.5.

**Fig. 8:**
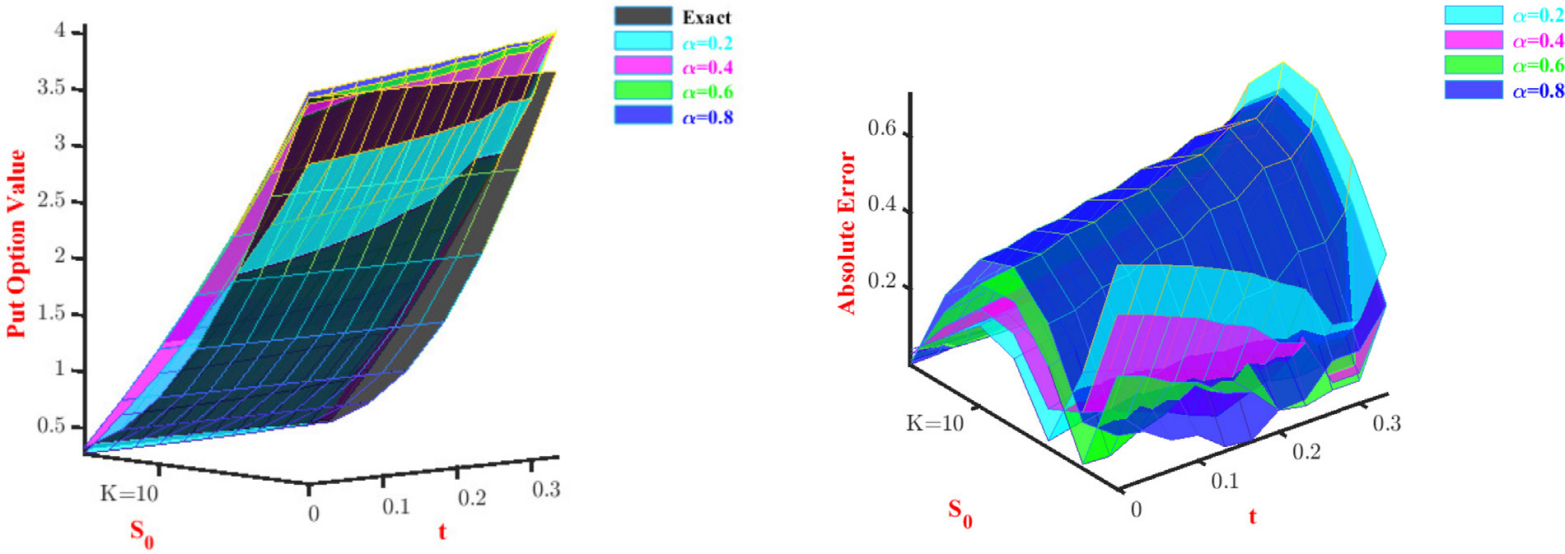
Prices and absolute errors to approximate Put option values for Problem-4 with β=1.6.

**Fig. 9:**
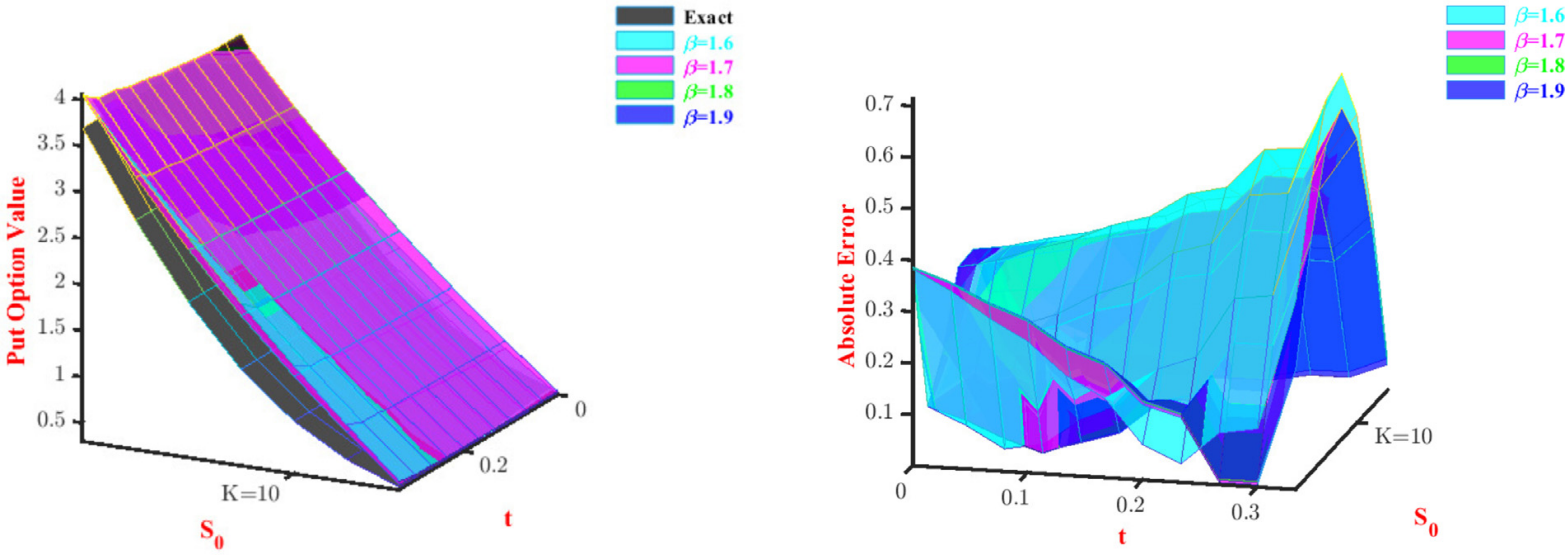
Prices and absolute errors to approximate Put option values for Problem-4 with α=0.5.

Table 15 and Table 16 display that if we half the time step size, then the absolute error converges according to, O(Cδt). Both price and absolute error are shown in 3D visual representation for the call option in Fig. 6 and Fig. 7 and for put option in Fig. 8 and Fig. 9. The representation has been done for fixed α and β, respectively.

**Table 15:** Maximum errors to approximate Call option value for Problem-4 with N=2.

α=0.75andβ=1.6		
δt	$L_{\infty }$ norm	Convergence order C(δt)
1/10	3.1197	-
1/20	3.1412	0.9931
1/40	3.1528	0.9963

**Table 16:** Maximum errors to approximate Put option value for Problem-4 with N=2.

α=0.75andβ=1.6		
δt	$L_{\infty }$ norm	Convergence order C(δt)
1/10	0.4006	-
1/20	0.3855	1.0392
1/40	0.3730	1.0335

## Conclusions

In this study, we have attempted to solve the fractional diffusion equation with both time and space variables. To achieve our goals, we have derived elaborately the formulations with the aid of the Galerkin weighted residual method, and the Galerkin-Finite difference method for space fractional order, and time and space fractional order, respectively. For the latter case, we have introduced a new technique that is the combination of the Galerkin residual method for space derivative terms while finite difference approximation is used for time derivative terms. We have verified our proposed techniques on some numerical examples. Different types of errors are compared with the results of the published articles as well. Our technique has been applied to well-known Black-Scholes model to find the values of European options. Our computed results are in good agreement with the analytical solutions. Finally, we may conclude that the proposed new technique may apply to other fractional parabolic PDEs in both time and space variables.

## Limitations

Not Applicable.

## Ethics statements

The authors must comply with the ethical guidelines of *MethodsX*. In addition, none of the authors conducted studies involving human participants or animals or any kind of social media platforms in the preparation of this article.

## Declaration of Interest

The authors declare that they have no known competing financial interests or personal relationships that could have appeared to influence the work reported in this paper.

## CRediT authorship contribution statement

**Md. Shorif Hossan:** Visualization, Methodology, Writing – original draft, Writing – review & editing. **Trishna Datta:** Conceptualization, Methodology, Formal analysis, Visualization, Writing – original draft, Writing – review & editing. **Md. Shafiqul Islam:** Supervision, Validation, Writing – review & editing, Investigation.

## Data Availability

No data was used for the research described in the article.
